# Association of First Employment Characteristics and Hospitalization in the Mayo Clinic Biobank

**DOI:** 10.1093/geroni/igab046.3564

**Published:** 2021-12-17

**Authors:** Paul Takahashi, Euijung Ryu, Nicole Larson, Gregory Jenkins, Kirt Christine, Kathleen Yost, Janet Olson

**Affiliations:** Mayo Clinic, Rochester, Minnesota, United States

## Abstract

Work history is associated with long term health outcomes We hypothesize that characteristics of the first work experience, such as age at first job and length of work (hereafter job) are associated with future risk of hospitalization. We further hypothesize that the length of work will be associated with hospitalization. We conducted a survey of adults >60 years using a nested case-control approach within the Mayo Clinic Biobank. We collected job related variables including age at job start, reason for ending, and length of time. To test associations between each variable and hospitalization, we used age and gender adjusted logistic regression models. Our study included 4,024 subjects: 1,801 cases and 2223 controls. The mean age at time of match was 77.3 years (SD 7.2 years) with 49.2% males. Older age at the first full-time job was associated with lower chance of hospitalization later in life (OR=0.81 [0.67, 0.97] for those who started the job over 22 compared to those started at 18 or less). Cases were more likely to have stopped working because of illness (OR=2.04 [95% CI 1.29,3.27]). Cases were less likely to have stopped working because of retirement (OR=0.82 [95% CI: 0.72, 0.93]). We found cases were employed with a slightly shorter time (20.5 yrs. (SD 16.6)) compared to controls (21.8 yrs. (SD 16.3)) (p=0.005). Cases started work earlier and stopped work more frequently because of illness/disability compared to controls. This could reflect educational attainment in controls. This study highlights work history as potential predictor of future hospitalization.

